# Association of HbA1c and utilization of internal mammary arteries with wound infections in CABG

**DOI:** 10.3389/fcvm.2024.1345726

**Published:** 2024-03-18

**Authors:** Tim Knochenhauer, Andreas Schaefer, Jens Brickwedel, Beate Reiter, Shiho Naito, Svante Zipfel, Yvonne Schneeberger, Hermann Reichenspurner, Bjoern Sill

**Affiliations:** Department of Cardiovascular Surgery, University Heart and Vascular Center Hamburg, University Hospital Hamburg-Eppendorf, Hamburg, Germany

**Keywords:** HbA1c, coronary artery bypass grafting, coronary artery disease, wound healing disorder, deep sternal wound infection

## Abstract

**Background:**

Deep sternal wound infection (DSWI) remains a serious complication after coronary artery bypass grafting (CABG). We herein aimed to stratify diabetic patients who underwent CABG using bilateral internal mammary artery (BIMA) for levels of glycated hemoglobin A1C (HbA1c) and compare postoperative outcomes.

**Methods:**

Between January 2010 and August 2020, 4,186 consecutive patients underwent isolated CABG at our center. In 3,229 patients, preoperative HbA1c levels were available. Primary endpoints were wound healing disorder (WHD), DSWI, and 30-day mortality. Patients were stratified according to preoperative HbA1c levels. Patients were further divided into subgroups according to utilization of BIMA.

**Results:**

After adjustment, no differences in mortality and stroke rates were seen between group 1 (HbA1c < 6.5%) vs. group 2 (HbA1c ≥ 6.5%). WHD was more frequent in group 2 [2.8 vs. 5.6%; adjusted *p* = 0.002; adjusted odds ratio (OR), 1.853 (1.243–2.711)] but not DSWI [1.0 vs. 1.5%; adjusted *p* = 0.543; adjusted OR, 1.247 (0.612–2.5409)]. BIMA use showed a higher rate of WHD [no BIMA: 3.0%; BIMA: 7.7%; adjusted *p* = 0.002; adjusted OR, 4.766 (1.747–13.002)] but not DSWI [no BIMA: 1.1%; BIMA: 1.8%; adjusted *p* = 0.615; adjusted OR, 1.591 (0.260–9.749)] in patients with HbA1c ≥ 6.5%.

**Conclusions:**

Intraoperative utilization of BIMA is not connected with an increase of DSWI but higher rates of WHD in patients with poor diabetic status and HbA1c ≥ 6.5%. Therefore, application of BIMA should be taken into consideration even in patients with poor diabetic status, while identification of special subsets of patients who are at particular high risk for DSWI is of paramount importance to prevent this serious complication.

## Introduction

According to recent guidelines, for myocardial revascularization, coronary artery bypass grafting (CABG) is in particular recommended for treatment of complex coronary artery disease (CAD) in patients with intermediate to high SYNTAX (Synergy between Percutaneous Coronary Intervention with TAXUS and Cardiac Surgery) score and with type 2 diabetes (1, 2). Although results of randomized controlled trials and retrospective studies are partly contradictory, there is a wide consensus for utilization of the left (LIMA) and right internal mammary artery (RIMA), usually referred to as bilateral mammary artery (BIMA), in CABG since long-term patency rates were shown to be superior compared to saphenous vein grafts or the radial artery (3, 4). Especially patients with diabetes mellitus (DM) and reduced left ventricular function benefit from the utilization of BIMA during CABG in terms of long-term survival and freedom from re-revascularization (5, 6). However, a major concern regarding BIMA application in diabetic patients remains an increased risk of wound healing disorders (WHD) or deep sternal wound infections (DSWI) after CABG. Although WHD and DSWI after CABG are highly likely a multifactorial process including the impact on the patient's nutritional status, technique of IMA harvesting (skeletonized vs. pedicled), and/or the presence of metabolic syndrome and obesity (7–9), there are several reports suggesting a detrimental effect of BIMA usage in diabetic patients regarding WHD and DSWI (10, 11), while still resulting in decreased hospital mortality, cerebrovascular events, and re-revascularization rates (12). However, especially DSWI remains a serious complication after CABG with reported in-hospital mortality rates between 7% and 35% and a major socioeconomic impact (13), and therefore the identification of special subsets of patients who are at a particular high risk for DSWI is of utmost interest. So far, there is no report stratifying diabetic patients who underwent CABG using BIMA for levels of glycated hemoglobin A1C (HbA1c), as expression of adequate preoperative management of blood sugar levels. We herein aimed to perform this stratification at a high-volume tertiary heart center and correlate these subsets of diabetic patients with postoperative outcomes with a special emphasis on WHD and DSWI.

## Patients and methods

### Ethical statement

Data acquisition was performed anonymized and retrospectively. Therefore, in accordance with German law, no ethical approval is needed and informed patient consent was waived.

### Patients

**Figure 1:**
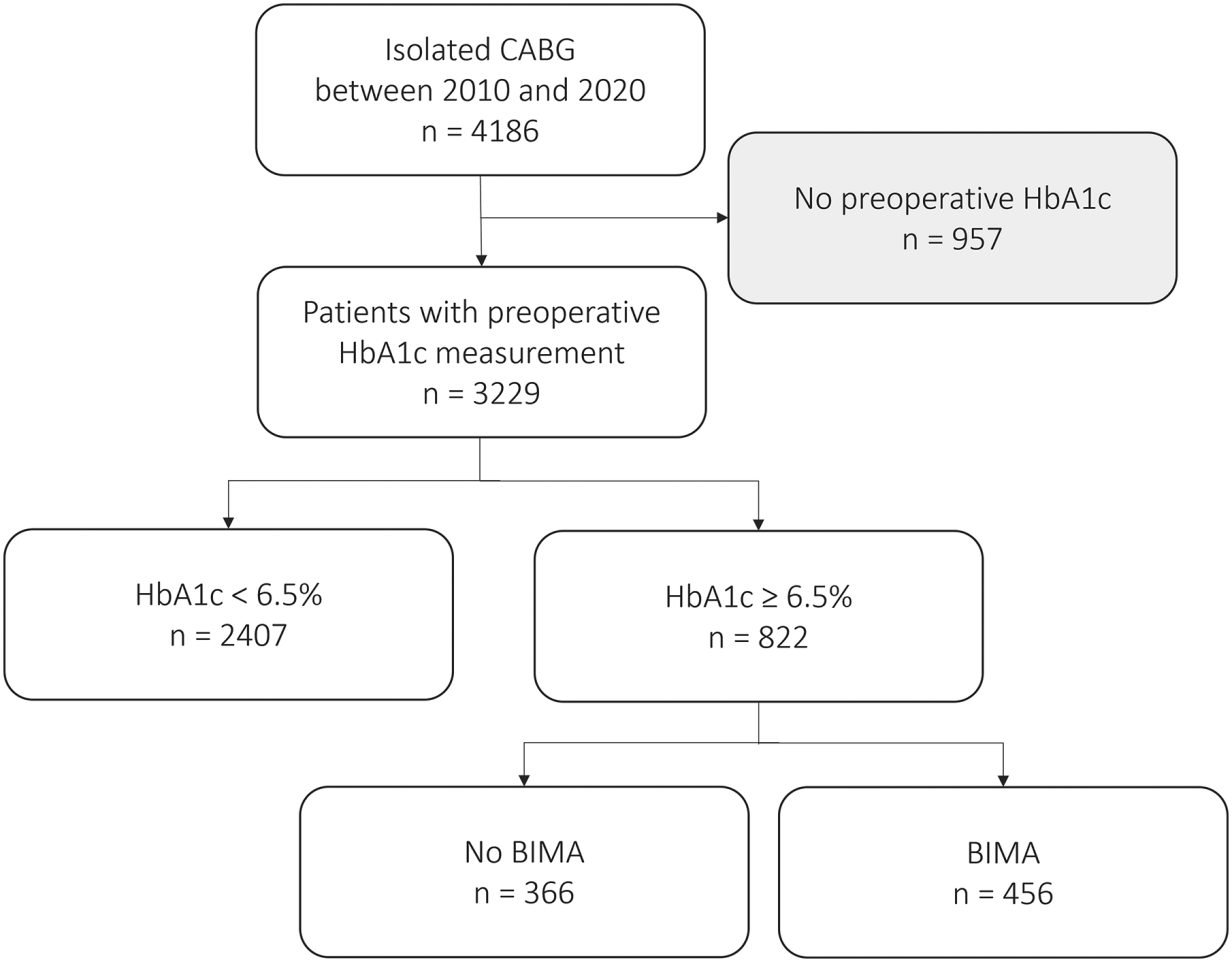
Patient inclusion flow chart.

Between January 2010 and August 2020, 4,186 consecutive patients underwent isolated CABG at our center. In 3,229 patients, preoperative HbA1c levels were available and retrospective inclusion of those patients into a dedicated database was performed. A patient inclusion flow chart is shown in Figure 1. Harvesting of IMA grafts was performed in a skeletonized fashion. Application of topical vancomycin paste prior to sternum closure was in use since 2017 in all patients. The general closure technique included utilization of sternal wires, inserted in a single or figure of eight fashion and closure of subcutaneous layers using running or single Vicryl sutures. Skin closure was performed with intracutaneous absorbable sutures or a skin stapler. Vacuum therapy was conducted using the KCI system (Kinetic Concepts Inc., San Antonio, TX, USA).

Primary endpoints for this study were WHD, DSWI, and 30-day mortality. Secondary endpoints comprised of major adverse cardiac and cereborvascular events (MACCE; stroke, myocardial infarction) and postoperative renal failure and sepsis. Patients were stratified according to preoperative HbA1c levels with the standard cut-off value of 6.5% and definition of poor diabetic status in patients with HbA1c > 6.5% (14). DSWI was defined as postoperative infection involving the sternum and mediastinal space. WHD was defined as wound infection involving the subcutaneous layer without involvement of the sternum or mediastinal space or as inappropriate healing of the subcutaneous layer due to malperfusion of tissue without signs of infection.

Patients with poor diabetic status were further divided into subgroups according to the utilization of BIMA during CABG. The absence of BIMA use was defined as the application of single IMA, solely veins, single IMA + radial artery or the combination of single IMA + vein.

### Statistical analyses

Continuous variables are shown as mean ± standard deviation and are compared using the Mann–Whitney *U*-test. Binary variables are shown as counts (frequencies) and are compared using the *χ*^2^ test. Logistic regression was performed to identify the odds ratio (OR) for outcome parameters. Procedural and outcome parameters were adjusted for body mass index (BMI), left ventricular ejection fraction (LVEF) <35%, prior stroke, extent of CAD, European System for Cardiac Operative Risk Evaluation II (EuroSCORE II), prior CABG, HbA1c, and extracardiac arteriopathy.

A *p*-value of <0.05 was considered statistically significant. All analyses were performed with SPSS statistical software version 26 (IBM Inc., Armonk, NY, USA).

## Results

### Baseline demographics

Patients with HbA1c ≥ 6.5% (group 1) showed a significant comorbidity burden less frequently compared to patients with HbA1C > 6.5% (group 2). In particular, patients with a poor diabetic status presented preoperatively with a higher body mass index (27.63 ± 5.37 vs. 29.52 ± 4.79 kg/m^2^; *p* < 0.001), more frequent severely reduced left ventricular function (left ventricular ejection fraction <35%: 4.7% vs. 7.5%; *p* = 0.002), more prior strokes (7.7% vs. 12.3%; *p* < 0.001), a greater extent of CAD (number of diseased vessels: 2.46 ± 1.0 vs. 2.58 ± 0.93; *p* < 0.001), prior CABG (0.5% vs. 1.2%; *p* = 0.046), and a higher rate of extracardiac arteriopathy (17.3% vs. 22.6%; *p* = 0.001). Overall, this resulted in a higher EuroSCORE II in patients with a poor diabetic status (1.38% ± 1.16% vs. 1.69% ± 1.86%; *p* < 0.001). Further baseline characteristics showed no significant differences between both groups.

Detailed patient demographics are summarized in Table 1.

**Table 1:** Baseline data.

	HbA1c < 6.5% (*n* = 2,407)	HbA1c ≥ 6.5% (*n* = 822)	*p*-value
Age, years	67.6 (±9.7)	67.7 (±9.5)	0.827
Male gender, *n* (%)	1,982 (82.3)	670 (81.5)	0.590
STEMI, *n* (%)	156 (6.5)	41 (5.0)	0.124
BMI, kg/m^2^	27.6 (±5.4)	29.5 (±4.8)	<0.001
COPD^a^, *n* (%)	164 (6.8)	62 (7.5)	0.479
LVEF < 35%, *n* (%)	112 (4.7)	62 (7.5)	0.002
Prior stroke, *n* (%)	185 (7.7)	101 (12.3)	<0.001
Creatinine clearance	86.5 (±30.9)	88.0 (±35.2)	0.254
Dialysis, *n* (%)	24 (1.0)	13 (1.6)	0.174
Number of diseased vessels	2.5 (±1.0)	2.6 (±0.9)	0.003
EuroSCORE II, %	1.4 (±1.2)	1.7 (±1.9)	<0.001
Prior CABG, *n* (%)	13 (0.5)	10 (1.2)	0.046
Prior PCI, *n* (%)	461 (19.2)	174 (21.2)	0.327
Extracardiac arteriopathy^b^, *n* (%)	417 (17.3)	186 (22.6)	0.001
NYHA ≥ III, *n* (%)	972 (40.4)	620 (75.4)	0.114

STEMI, ST-elevation myocardial infarction; COPD, chronic obstructive pulmonary disease; PCI, percutaneous coronary intervention; NYHA, New York Heart Association.

^a^ Extracardiac arteriopathy.

^b^ COPD according to EuroSCORE definitions.

### Periprocedural data

In patients with a poor diabetic status, a less frequent use of BIMA grafting was seen (60.3% vs. 55.5%; *p* = 0.016). Furthermore, in the early postoperative stage, these patients were more frequently prone to prolonged ventilation >24 h (3.0% vs. 5.5%; *p* = 0.001) and a prolonged need for inotropes >24 h (2.6% vs. 4.0%; *p* = 0.041). Majority of patients in both groups were referred for CABG as elective procedures. No statistically significant differences between groups were seen regarding the applied intraoperative techniques (off-pump coronary artery bypass, skeletonized harvesting of internal mammary arteries), number of performed bypasses, and documented procedure times including time of extracorporeal circulation and aortic cross-clamp time.

Detailed periprocedural data are summarized in Table 2.

**Table 2:** Periprocedural data.

	HbA1c < 6.5% (*n* = 2,407)	HbA1c ≥ 6.5% (*n* = 822)	*p*-value
Elective procedure, *n* (%)	2,078 (86.3)	710 (86.4)	0.975
OPCAB, *n* (%)	1,373 (57.0)	458 (55.7)	0.508
Skeletonized IMA harvesting, *n* (%)	2,055 (85.4)	719 (87.5)	0.222
Procedure time, min	260.4 (±76.3)	265.4 (±77.4)	0.104
ECC, min	68.8 (±62.6)	70.4 (±65.3)	0.534
ACC, min	45.0 (±43.8)	44.6 (±44.7)	0.832
Number of bypasses, n	2.46 (±0.79)	2.49 (±0.80)	0.474
BIMA grafting, *n* (%)	1,451 (60.3)	456 (55.5)	0.016
Prolonged inotropes ≥24 h, *n* (%)	63 (2.6)	33 (4.0)	0.041
Prolonged ventilation ≥24 h, *n* (%)	73 (3.0)	45 (5.5)	0.001
Extracorporeal circulation support, *n* (%)	48 (1.9)	21 (2.6)	0.588

ECC, extracorporeal circulation time; ACC, aortic cross-clamp time; IMA, internal mammary artery; BIMA, bilateral internal mammary artery; OPCAB, off-pump coronary artery bypass.

### Thirty-day outcome parameters

In unadjusted analysis, patients with a poor diabetic status presented with a higher rate of WHD (2.8% vs. 5.6%; *p* < 0.001) but not DSWI (1.0% vs. 1.5%; *p* = 0.3). These patients also presented a higher mortality rate (0.9% vs. 2.1%, *p* = 0.006), more frequent stroke (1.5% vs. 2.8%, *p* = 0.037), more frequent renal failure (5.6% vs. 8.5%, *p* = 0.025), and a prolonged postoperative intensive care unit (ICU) stay (2.52 ± 2.59 vs. 3.08 ± 5.60 days; *p* < 0.001).

However, after adjusting for differing baseline characteristics, no differences regarding 30-day mortality and stroke rates were seen. The analysis showed a higher rate of WHD in patients with an HbA1c higher than 6.5% [2.8% vs. 5.6%; adjusted *p* = 0.002; adjusted OR, 1.853 (1.243–2.711)] without significant differences with regard to DSWI [1.0% vs. 1.5%; adjusted *p* = 0.543; adjusted OR, 1.247 (0.612–2.5409)]. Patients with a poor diabetic status still presented a higher rate of 30-day renal failure [5.6% vs. 8.5%; adjusted *p* = 0.042; adjusted OR, 1.373 (1.012–1.864)] and a longer ICU stay [2.52 ± 2.59 vs. 3.08 ± 5.60 days; adjusted *p* = 0.006; adjusted OR, 0.405 (0.115–0.695)]. No differences regarding 30-day myocardial infarction or sepsis were seen between groups in unadjusted and adjusted analysis. When stratifying WHD/DSWI according to the different levels of HbA1c, a significant increase of WHD/DSWI was seen in patients with HbA1c > 9% even when compared to patients with HbA1c > 6% (HbA1c 5–5.9% vs. >9%: WHD/DSWI 2.5% vs. 15%; *p* < 0.001; HbA1c 6–6.9% vs. >9%: WHD/DSWI 2.5% vs. 4.5%; *p* = 0.01).

**Figure 2:**
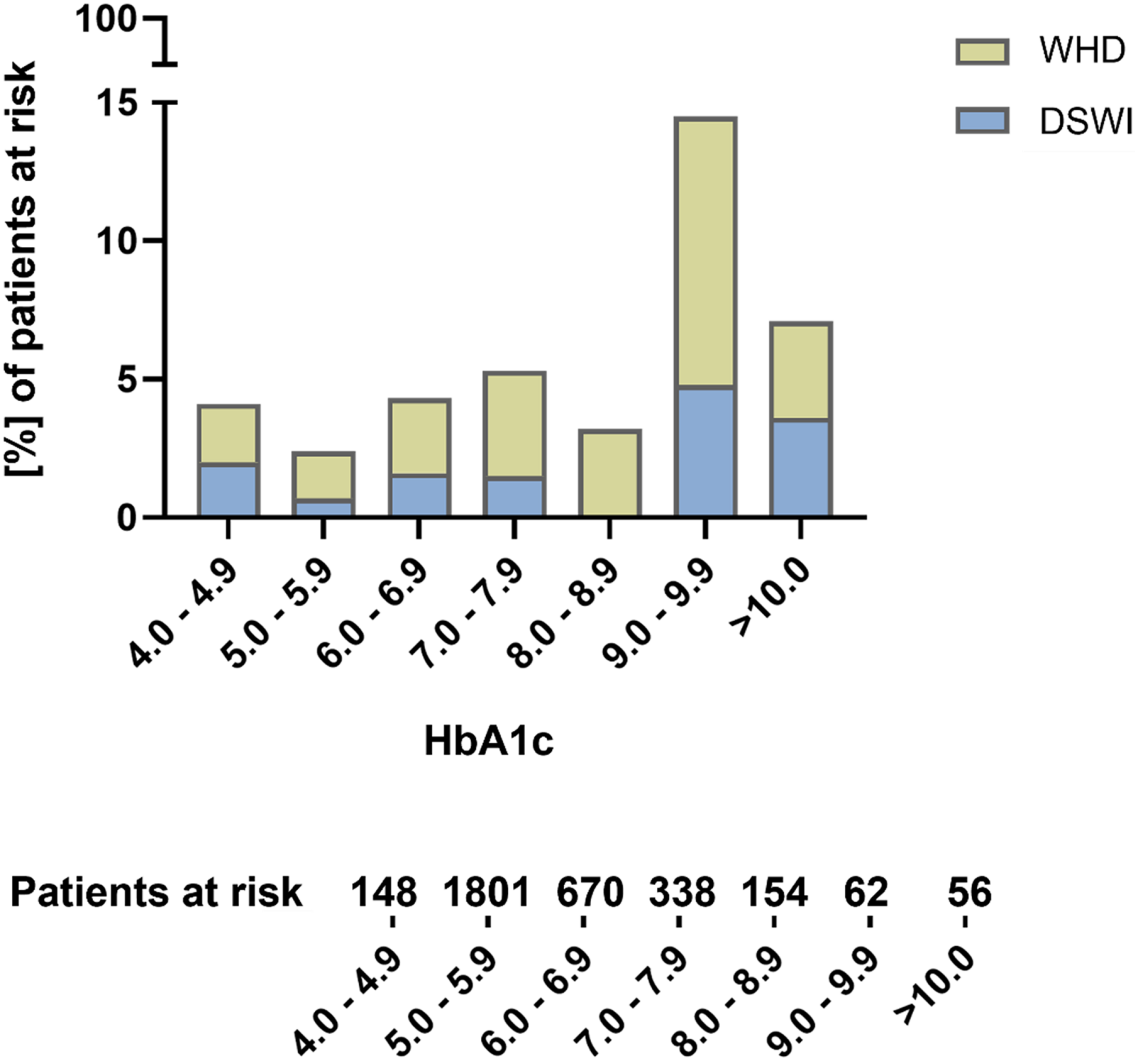
Distribution of wound healing disorders and deep sternal wound infections according to preoperative HbA1c levels.

Detailed 30-day outcome parameters are summarized in Table 3. The distribution of WHD and DSWI according to HbA1c levels is shown in Figure 2.

**Table 3:** 30-day outcome parameter.

	HbA1c < 6.5% (*n* = 2,407)	HbA1c ≥ 6.5% (*n* = 822)	Unadjusted *p*-value	Adjusted OR (95% CI)	Adjusted *p*-value
WHD, *n* (%)	68 (2.8)	46 (5.6)	<0.001	1.835 (1.243–2.711)	0.002
DSWI, *n* (%)	24 (1.0)	13 (1.5)	0.301	1.247 (0.612–2.540)	0.543
ICU stay, days	2.52 (±2.59)	3.08 (±5.60)	<0.001	0.405 (0.115–0.695)	0.006
Hospital stay, days	8.44 (±15.98)	9.95 (±28.9)	0.063	1.404 (−0.224–3.033)	0.091
Myocardial infarction, *n* (%)	48 (2.0)	15 (1.8)	0.804	0.818 (0.436–1.537)	0.533
Mortality, *n* (%)	21 (0.9)	17 (2.1)	0.006	1.753 (0.878–3.500)	0.111
Stroke, *n* (%)	35 (1.5)	23 (2.8)	0.037	1.684 (0.975–2.908)	0.062
Renal failure, *n* (%)	134 (5.6)	70 (8.5)	0.025	1.373 (1.012–1.864)	0.042
Sepsis, *n* (%)	14 (0.6)	11 (1.3)	0.086	1.526 (0.648–3.594)	0.334

WHD, wound healing disorder; DSWI, deep sternal wound infection; ICU, intensive care unit.

### Spectrum of pathogens and antimicrobial therapy in patients with WHD and DSWI (HbA1c < 6.5% vs. HbA1c ≥ 6.5%)

**Figure 3:**
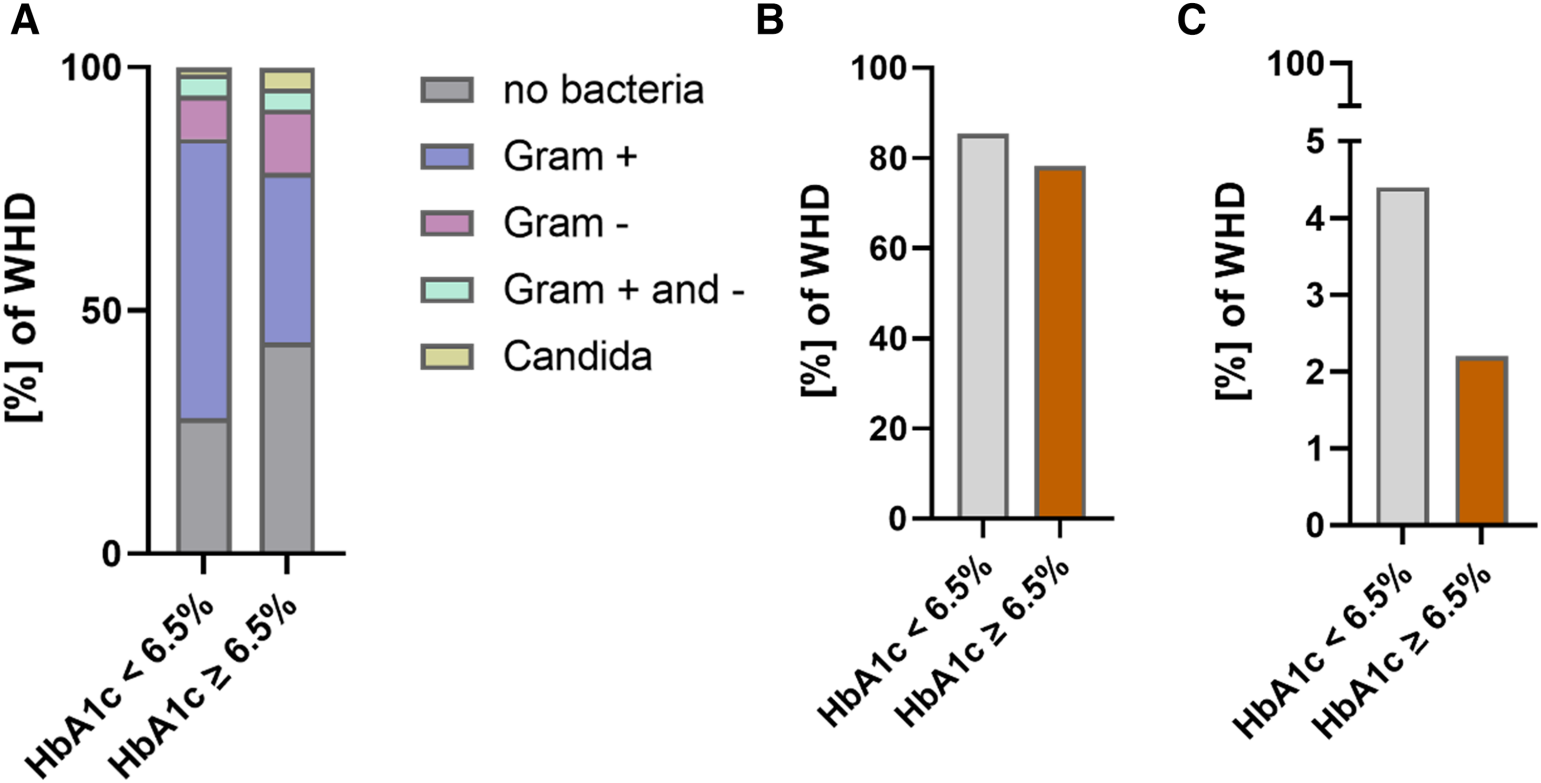
Bacteria and antimicrobial treatment in patients with WHD (HbA1c < 6.5% vs. HbA1c ≥ 6.5%). (**A**) Bacterial species in patients with WHD. (**B**) Proportion of antimicrobial treatment in patients with WHD. (**C**) Percentage of resistant bacteria in patients with WHD.

There were no significant differences between groups regarding the distribution of causal pathogens for WHD and DSWI with a large proportion of patients in both groups with no evidence of any bacterial wound infection. Most frequent pathogens were gram + species in both groups followed by gram − species. The proportion of antimicrobial treatment was not different between groups, whereas a higher but not statistically significant proportion of patients with HbA1c < 6.5% presented with resistant bacteria in microbiological culturing of intraoperative tissue specimen. The rate of antibiotic treatment and type of bacteria in patients with HbA1c < 6.5% vs. HbA1c ≥ 6.5% are shown in Figure 3.

### Subgroup analysis: WHD and DSWI in patients with BIMA use and HbA1c ≥ 6.5%

**Figure 4:**
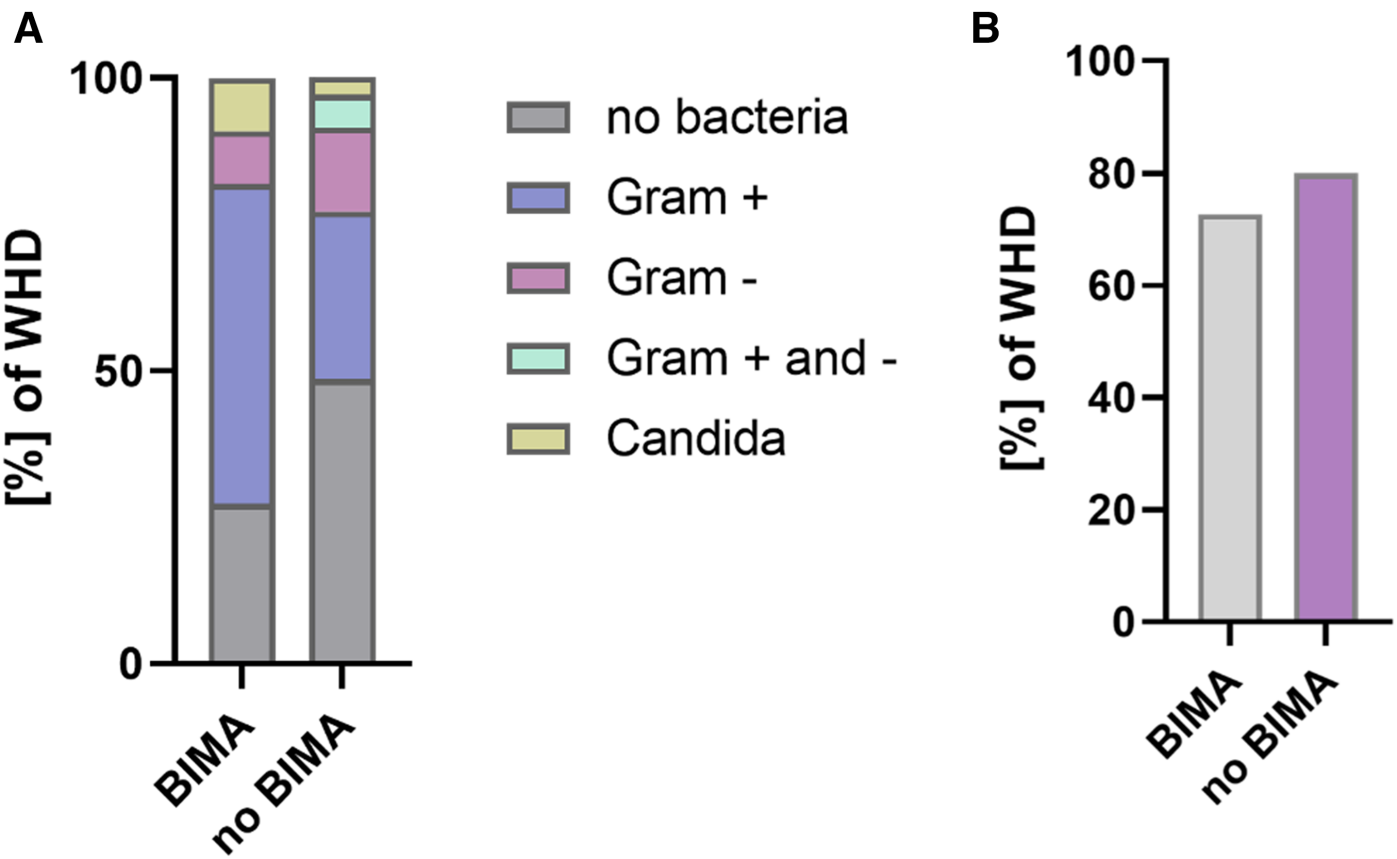
Bacteria and antimicrobial treatment in WHD/DSWI (BIMA vs. no BIMA in patients with HbA1c ≥ 6.5%). (**A**) Bacterial species in patients with WHD. (**B**) Proportion of antimicrobial treatment in patients with WHD.

In a subgroup analysis of patients with a poor diabetic status and comparison of BIMA vs. no BIMA use, BIMA use showed a higher rate of WHD [no BIMA: 3.0%; BIMA: 7.7%; adjusted *p* = 0.002; adjusted OR, 4.766 (1.747–13.002)] but no difference in the rate of DSWI [no BIMA: 1.1%; BIMA: 1.8%; adjusted *p* = 0.615; adjusted OR, 1.591 (0.260–9.749)]. There was no significant difference in 30-day MACCE between no BIMA and BIMA use in patients with an HbA1c ≥ 6.5%. The rate of antibiotic treatment and type of bacteria in patients with BIMA use vs. no BIMA use are shown in Figure 4. Since higher age was described as a potential risk factor for postoperative WHD and DSWI, an additional logistic regression analysis was performed to determine the significance of age as a risk factor for WHD and DSWI. Here, age presented no significance (see Supplementary Table S1).

Detailed baseline characteristic subgroups and rates of WHD and DSWI in patients with BIMA use are summarized in Tables 4, 5.

**Table 4:** Baseline data subgroup analysis: BIMA use in patients with HbA1c ≥ 6.5%.

	no BIMA (*n* = 366)	BIMA (*n* = 456)	*p*-value
Age, years	71.8 (±9.0)	64.3 (±8.6)	<0.001
Male gender, *n* (%)	272 (74.3)	398 (87.3)	<0.001
STEMI, *n* (%)	20 (5.5)	21 (4.6)	0.579
BMI, kg/m^2^	29.5 (±5.2)	29.6 (±4.4)	0.791
COPD^a^, *n* (%)	37 (10.1)	25 (5.5)	0.013
LVEF < 35%, *n* (%)	32 (8.7)	30 (6.6)	0.243
Prior stroke, *n* (%)	49 (13.4)	52 (11.4)	0.389
Creatinine clearance	76.6 (±30.7)	97.2 (±36.0)	<0.001
Dialysis, *n* (%)	7 (1.9)	6 (1.3)	0.495
Number of diseased vessels, *n*	2.5 (±1.0)	2.6 (±0.9)	0.173
EuroSCORE II, %	2.3 (±2.5)	1.2 (±0.9)	<0.001
Prior CABG, *n* (%)	8 (2.2)	2 (0.4)	0.023
Prior PCI, *n* (%)	86 (23.5)	88 (19.3)	0.143
Extracardiac arteriopathy^b^, *n* (%)	88 (24.0)	98 (21.5)	0.385
NYHA ≥ III, *n* (%)	175 (47.8)	171 (37.5)	0.001

STEMI, ST-elevation myocardial infarction; COPD, chronic obstructive pulmonary disease; PCI, percutaneous coronary intervention; NYHA, New York Heart Association.

^a^ Extracardiac arteriopathy.

^b^ COPD according to EuroSCORE definitions.

**Table 5:** Thirty-day outcomes subgroup analysis: BIMA use in patients with HbA1c ≥ 6.5%.

	no BIMA (*n* = 366)	BIMA (*n* = 456)	*p*-value	Adjusted OR (95% CI)	Adjusted *p*-value
WHD, *n* (%)	11 (3.0)	35 (7.7)	0.004	4.766 (1.747–13.00)	0.002
DSWI, *n* (%)	4 (1.1)	8 (1.8)	0.374	1.591 (0.260–9.749)	0.615
Myocardial infarction, *n* (%)	10 (2.7)	5 (1.1)	0.082	0.980 (0.206–4.649)	0.979
Mortality, *n* (%)	11 (3.0)	6 (1.3)	0.091	1.689 (0.383–6.123)	0.489
Stroke, *n* (%)	11 (3.0)	12 (2.6)	0.747	1.257 (0.407–3.877)	0.691
Renal failure, *n* (%)	41 (11.2)	31 (6.8)	0.026	1.035 (0.518–2.066)	0.923
Sepsis, *n* (%)	7 (1.9)	4 (0.9)	0.199	1.302 (0.239–7.106)	0.761

WHD, wound healing disorder; DSWI, deep sternal wound infection.

## Comment

Main findings of the herein conducted study are (I) in adjusted analysis, CABG in patients with a poor diabetic status is not associated with an increase in mortality, stroke, or DSWI while presenting a higher rate of WHD and renal failure compared to patients with HbA1c < 6.5%; (II) in our cohort, BIMA was significantly less applied in patients with poor diabetic status; (III) most WHD and DSWI were seen in patients with HbA1c > 9%; and (IV) BIMA utilization in patients with HbA1c ≥ 6.5% presents excellent short-term results, largely without an increase in primary or secondary endpoints and in particular no increase of DSWI while yielding a higher rate of WHD.

While several analyses suggest a detrimental effect of elevated HbA1c levels in patients undergoing CABG with regard to postoperative mortality, stroke, and myocardial infarction (15, 16), our results presented no increase in the mentioned clinical endpoints. However, with higher rates of renal failure in patients with HbA1c ≥ 6.5%, the results presented herein are in accordance with the work of Oezkur et al. (17) who reported that in 307 patients undergoing CABG chronic hyperglycemia was independently associated with postoperative acute kidney injury. Reasons for these partly contradictory results remain speculative but the herein documented high rates of elective procedures and utilization of off pump coronary artery bypass (OPCAB) technique in more than 50% of patients in both groups may have contributed to the low rates of stroke and postoperative myocardial infarction seen herein, since OPCAB is considered to potentially reduce stroke rates in CABG (18) and elective procedures are connected with lower rates of mortality and myocardial infarction compared to emergency CABG as seen in larger registries (19). While WHD were more frequent in patients with a poor diabetic status, DSWI presented no significant increase in the patient cohort investigated herein. This effect persisted in adjusted analysis and in comparison of BIMA utilization in patients with HbA1c ≥6.5% and ≥6.5%. Contrary to our results, the E-CABG registry reported a clear adverse effect of poor diabetic status on postoperative rates of DSWI in CABG (20), while it has to be emphasized that in the mentioned work, the chosen HbA1c cut-off was different (4.6%; 70 mmol/mol) to the herein used cut-off value, which may partly explain the differences in the results. Furthermore, a meta-analysis from Dai et al. presented a detrimental effect of BIMA utilization in diabetic patients with regard to postoperative DSWI (21), an effect that was also not seen in the patients investigated herein and which may be caused by lack of adjustment for preoperative risk factors and diffuse definition and separation of WHD and DSWI in the included 32 studies. Although retrospectively conducted, our work conclusively showed that patients with poor diabetic status and patients with poor diabetic status provided with BIMA during CABG are not prone to increased rates of DSWI. Measures that may have contributed to the described low rates of DSWI may be the routine use of topical vancomycin paste (22) and high rates of skeletonized IMA harvesting (23), techniques that are considered to reduce the risk of DSWI. When considering advantages of BIMA in patients with diabetes, which are mainly improved long-term survival and reduced re-revascularization rates (5), our results suggest that BIMA utilization in all diabetic patients and especially also in patients with poor diabetic status and preoperative HbA1c ≥ 6.5% is reasonable, since DSWI were not increased. Although, a higher rate of superficial WHD was seen, the mentioned benefits of BIMA should outweigh the drawbacks of WHD, which are usually simple to treat.

However, our results suggest that special attention should be paid to patients with HbA1c > 9%, since a clear trend toward a significant increase of WHD and DSWI was seen and benefits of BIMA should be weighed against possible complications connected with DSWI. Therefore, further analysis for the identification of patient subsets that are at particular high risk for DSWI after CABG and in particular after CABG using BIMA are required to further reduce risk of DSWI, which remains a serious and harmful complication after cardiac surgery.

### Limitations

Limitations are inherent in the retrospective, single-center study design with limited patient numbers: patients were not randomized to a specific treatment; therefore, patient preselection with hidden confounders may apply. Moreover, data on preoperative medication (e.g., immunosuppressant drugs) that may influence wound healing were not available.

## Conclusions

CABG presents excellent short-term outcomes in terms of mortality, stroke, and myocardial infarction independent from preoperative HbA1c levels with no increase of DSWI, but higher rates of WHD and renal failure in patients with poor diabetic status and HbA1c ≥ 6.5%. Intraoperative utilization of BIMA is not connected with an increase of DSWI but higher rates of WHD in patients with poor diabetic status and HbA1c ≥ 6.5%. Therefore, the application of BIMA should be taken into consideration in CABG even in patients with poor diabetic status, while the identification of special subsets of patients who are at particular high risk for DSWI is of paramount importance to prevent this serious complication.

## Data Availability

The raw data supporting the conclusions of this article will be made available by the authors, without undue reservation.
