# Supplementary material for: Association of HbA1c and utilization of internal mammary arteries with wound infections in CABG
Source: Front Cardiovasc Med. 2024 Mar 18;11:1345726. doi: 10.3389/fcvm.2024.1345726 (PMC10982407; doi:10.3389/fcvm.2024.1345726)
Supplement: Supplementary file 1 [file Table1.docx]

# Supplement 1: Logistic regression analysis including age

# Table 1. Logistic regression analysis for wound healing disorder including age

|  | **OR** | **95% CI** | **p value** |
| --- | --- | --- | --- |
| Age [years] |  |  | 0.946 |
| Male gender | 0.262 | 0.175, 0.391 | **< 0.001** |
| BMI [kg/m²] | 1.035 | 1.014, 1.056 | **0.001** |
| Redo surgery |  |  | 0.871 |
| HbA1c [%] | 1.288 | 1.136, 1.460 | **< 0.001** |
| BIMA | 0.399 | 0.250, 0.638 | **< 0.001** |

^BMI: body mass index, BIMA: bilateral internal mammary artery, HbA1c: glycated hemoglobin A1c^

# Table 2. Logistic regression analysis for deep sternal wound infection including age

|  | **OR** | **95% CI** | **p value** |
| --- | --- | --- | --- |
| Age [years] |  |  | 0.304 |
| Male gender | 0.420 | 0.205, 0.859 | **0.017** |
| BMI [kg/m²] | 1.031 | 1.005, 1.057 | **0.017** |
| Redo surgery |  |  | 0.998 |
| HbA1c [%] | 1.246 | 1.002, 1.549 | **0.048** |
| BIMA |  |  | 0.334 |

^BMI: body mass index, BIMA: bilateral internal mammary artery, HbA1c: glycated hemoglobin A1c^
